# Prevention of type 2 diabetes mellitus with acupuncture: Protocol for a systematic review and meta-analysis

**DOI:** 10.1097/MD.0000000000013355

**Published:** 2018-11-30

**Authors:** Liwei Shi, Ling Feng, Yanan Yang, Xiaowen Li, Meizhen Zhang, Yueying Zhang, Qing Ni

**Affiliations:** a Department of Endocrinology; b Department of Health Care, Guang’an Men Hospital, China Academy of Chinese Medical Sciences; c Beijing University of Chinese Medicine, Beijing, China.

**Keywords:** acupuncture, diabetes prevention, meta-analysis, protocol, systematic review

## Abstract

**Background:**

Prediabetes is prevalent and significantly increases lifetime risk of progression to type 2 diabetes mellitus (T2DM). Acupuncture has been increasingly used for prediabetes in China but its effect is unclear. We aim to assess the efficacy and safety of acupuncture in preventing or delaying incident diabetes among individuals with prediabetes.

**Methods:**

The following 8 databases will be searched from inception to September 1, 2018: the Cochrane Central Register of Controlled Trials (CENTRAL) on the Cochrane Library, PubMed, Embase, Chinese National Knowledge Infrastructure database, Chinese Biomedical Literature database, Chinese Scientific Journal database, Wan Fang database, and Clinical Trials. The incidence of diabetes and regression toward normoglycemia will be accepted as the primary outcomes. The Cochrane Risk of Bias tool will be used to evaluate the methodologic quality of eligible studies. Meta-analysis will be performed by Review Manager 5.3.

**Results:**

This study will provide a high-quality synthesis of current evidence of acupuncture in the prevention of T2DM from several aspects including the incidence of diabetes, regression toward normoglycemia, fasting plasma glucose, 2-hour plasma glucose level after a 75-g oral glucose tolerance test, glycosylated hemoglobin level, body mass index, and adverse drug events.

**Conclusions:**

The conclusion of this review will provide evidence to judge whether acupuncture is an effective and safe intervention for prediabetes.

**PROSPERO registration number:**

PROSPERO CRD42018111236.

## Abstract 2

Supplemental Digital Content is available in the text

## 1 Introduction

Diabetes mellitus is currently a major public health problem. The International Diabetes Federation estimates that 420 million adults worldwide now have diabetes and 318 million have impaired glucose tolerance (IGT), with a projected global prevalence of 642 and 482 million, respectively, by 2040.^[1,2]^ China has the world's largest diabetes and prediabetes epidemic, which continues to increase.^[3]^ The toll of diabetes and diabetic complications on patients’ health and quality of life is enormous. The burgeoning diabetes pandemic also comes with high personal and financial costs to the individual, society, and the economy. Prediabetes is an intermediate metabolic state between normoglycemia and diabetes, including either IGT, impaired fasting glucose (IFG), or both conditions.^[4]^ IGT and IFG are “high-risk” states for diabetes development.^[4,5]^ The risk of progression to diabetes is greater for patients with IGT and IFG close to the diagnostic boundary for diabetes. On average, about 15% to 30% of adults with prediabetes are expected to progress to diabetes in 5 years.^[6]^ From a population perspective, this risk is substantial given the high prevalence of prediabetes.

Accordingly, many studies have examined the efficacy of lifestyle modification (LM) and pharmacologic interventions in preventing or delaying incident diabetes among patients with prediabetes. LM has been shown to reduce the incidence of type 2 diabetes mellitus (T2DM), and is recommended for prediabetes by American Diabetes Association guidelines.^[7–9]^ However, it is not easy for patients with prediabetes to maintain rigorous and sustained LM for the long term. Numerous landmark trials in the past quarter-century have demonstrated that various pharmacologic interventions, including metformin, acarbose, pioglitazone, rosiglitazone, and orlistat, can effectively delay or prevent the development of T2DM in high-risk individuals with IGT.^[10–19]^ Despite the clarity of these findings, translational prevention programs have faced many real-world impediments, and none of the tested interventions have an approved label for use in the prevention of T2DM and have been widely adopted as components of routine clinical care.

Acupuncture, one of the important treatments used in traditional Chinese medicine (TCM), involves the insertion of fine needles into the skin at specific acupoints. It has a long history of use in China and is increasing accepted by people in the developing and developed world as an alternative therapy to conventional treatments. The selection of acupoints is based on the syndrome differentiation from the diagnosis according to TCM theory. Pattern identification is a unique TCM concept that summarizes and differentiates the nature, location, and pattern of diseases, which is the essential guide for TCM therapy. The precisely tailoring acupuncture acupoints for individuals based on each individual pattern type can maximize its efficacy. TCM emphasizes the idea that health is maintained by a balance of energy within the body. Acupuncture helps to correct imbalances to relieve symptoms and treat the disease by stimulating various acupoints based on individual syndrome differentiation.

Acupuncture has been widely used for the treatment of diabetes and diabetic complications in China. Previous studies indicated that acupuncture therapy might be effective for diabetes and its complications (e.g., diabetic gastroparesis, diabetic peripheral neuropathy).^[20–22]^ Mechanistic studies applying acupuncture to the treatment of diabetes suggest that the major effects are associated with the control of blood glucose, body weight loss, protection of pancreas islet function, improvement of insulin resistance, and adjustment of the levels of related hormones, such as melatonin, insulin, glucocorticold, and epinephrine.^[23,24]^ Moreover, acupuncture treatment combined with hypoglycemic drugs has a synergic effect in lowering blood glucose.^[23]^ Currently, acupuncture has been increasingly used in the prevention of T2DM. Some studies indicated that acupuncture could increase the regression toward normoglycemia, lose body weight, lower fasting plasma glucose (FPG), 2-hour plasma glucose (2-hPG), and glycosylated hemoglobin (HbA1c) in patients with prediabetes.^[25,26]^ However, few systematic reviews have been conducted to evaluate the effect of acupuncture in the prevention of T2DM. Therefore, we aim to systematically assess the efficacy and safety of acupuncture in preventing or delaying incident diabetes among individuals with prediabetes.

## 2 Methods

### 2.1 Inclusion criteria for study selection

#### 2.1.1 Types of studies

All randomized controlled trials (RCTs) of acupuncture for prediabetes will be included. Excluded from the meta-analysis are duplicated publications, studies with unavailable or incorrect data, articles not reporting outcomes of interest. Also excluded are studies enrolling fewer than 10 participants and quasi-randomized controlled clinical trials (i.e., allocation using alternation, the sequence of admission, case record numbers, and dates of birth). The articles will not be restricted based on publication type.

#### 2.1.2 Types of patients

Participants with prediabetes, including IFG, IGT, or both conditions, will be included irrespective of gender, age, and ethnicity. All patients must be diagnosed with prediabetes by clearly defined or internationally recognized criteria.

#### 2.1.3 Types of interventions

Acupuncture type can include: body acupuncture, electroacupuncture, ear acupuncture, scalp acupuncture, fire needling, plum blossom needle, elongated needle, intradermal needling, or dry needling. The control intervention can include: no treatment, placebo/sham acupuncture, or other interventions (e.g., drugs, LM). Trials that evaluate acupuncture plus another therapy compared with the same therapy alone will also be included. Trials that only compare different types of acupuncture or different points will be excluded.

#### 2.1.4 Types of outcome measures

##### 2.1.4.1 The primary outcomes

The incidence of diabetes and regression toward normoglycemia will be assessed as the primary outcomes.

##### 2.1.4.2 The secondary outcomes

The secondary outcomes of this review will include FPG, 2-hPG level after a 75-g oral glucose tolerance test (OGTT), HbA1c level, body mass index (BMI), and adverse drug events.

### 2.2 Search methods for the identification of studies

#### 2.2.1 Electronic searches

The Cochrane Central Register of Controlled Trials (CENTRAL) on the Cochrane Library, PubMed, Embase, Chinese National Knowledge Infrastructure (CNKI) database, Chinese Biomedical Literature database, Chinese Scientific Journal database (VIP), and Wan Fang database will be searched to identify eligible trials published from inception to September 1, 2018. Ongoing registered clinical trials will be searched in the Clinical Trials, gov (https://www.Clinicaltrials.gov/). The search will be performed in English and Chinese. The following search terms will be used: acupuncture, acupuncture therapy, acupuncture treatment, acupoints, electroacupuncture, ear acupuncture, auricular acupuncture, scalp acupuncture, fire needling, plum blossom needle, elongated needle, intradermal needling, dry needling, prediabetes, impaired fasting glucose (IFG), impaired glucose tolerance (IGT), impaired glucose regulation (IGR), diabetes prevention, RCT, controlled clinical trial, randomized, placebo, drug therapy, randomly, trial, groups. The search terms will be translated into Chinese when reviewers search the Chinese databases. The search strategy for PubMed will be presented in supplementary file 1 (which describes the PubMed search strategy) and modified by using other databases.

#### 2.2.2 Searching other resources

The reference lists of studies and relevant systematic reviews will be examined for additional trials. The following literature sources in Chinese will also be searched: dissertations in CNKI, and conference papers in the China Conference Paper Database. Potential gray literatures will be searched in OpenGrey.eu.

### 2.3 Data collection and analysis

#### 2.3.1 Selection of studies

Records from electronic databases and other resources will be uploaded to a database created by NoteExpress v3.2.0.7103 software. The abstracts of all studies will be independently screened by the review authors (YY and XL). The full text of articles potentially suitable for the review will be obtained for further assessing eligibility based on the inclusion criteria or/and exclusion criteria. The studies that do not fulfill the inclusion criteria will be excluded and listed with reasons for their exclusion. Any disagreement will be resolved by consensus or discussion with a 3rd party (QN).

#### 2.3.2 Data extraction and management

Two review authors (YY and XL) will independently extract information on patients, methods, interventions, outcomes, and results using a data extraction form designed for this review. Extracted data will be compared by 2 review authors for completeness and accuracy and double-checked by another review author if necessary. Authors will be contacted by e-mail to obtain further data and verify methodologic quality when necessary. Any disagreement will be settled by discussion or by consulting a 3rd author (QN). The data extraction form will include the following items:

1. general information: title, authors, year of publication, and source;
2. trial characteristics: design, duration of follow-up, method of randomization, allocation concealment, incomplete outcome data, blinding (patients, people administering treatment, outcome assessors);
3. intervention(s): intervention(s) (type of acupuncture therapy, number and name of acupoints, duration of session), comparison intervention(s) (no treatment, placebo/sham therapy or other active treatment);
4. patients: total number and number in both groups, baseline characteristics, diagnostic criteria, withdrawals and losses to follow-up (reasons, description);
5. outcomes: outcomes specified above, length of follow-up, quality of reporting of outcomes.

#### 2.3.3 Assessment of risk of bias in included studies

The methodologic quality of RCTs will be assessed using each item specified by the Cochrane Risk of Bias Tool,^[27]^ including random sequence generation (selection bias), allocation concealment (selection bias), blinding of participants and personnel (performance bias), blinding of outcome assessment (detection bias), incomplete outcome data (attrition bias), selective reporting (reporting bias), and other bias. For each domain, the following description will be used to assess proper management of the risk of bias: low risk, high risk, or unclear. Each study will be respectively categorized as “low risk of bias” (all the items are in low risk), “high risk of bias” (1 or more items are in high risk), and “unclear risk of bias” (1 or more items are in unclear risk). Low risk of bias represents a good quality. Any disagreements will be resolved by discussion with QN.

#### 2.3.4 Measures of treatment effect

Dichotomous outcomes will be presented as risk ratio and 95% confidence intervals (CIs), and continuous outcomes will be presented as mean difference/standard mean difference and 95% CIs.

#### 2.3.5 Unit of analysis issues

The unit of analysis will be each patient recruited into the trials.

#### 2.3.6 Dealing with missing data

For each included study, the number of dropouts, exclusions from the analysis and missing data will be gathered by contacting the study author. If unable to obtain sufficient data, available case analysis will be performed. The potential impact of missing data on the findings of the review will be described in the discussion section if necessary.

#### 2.3.7 Assessment of heterogeneity

Statistical heterogeneity will be assessed with the *I*-square (*I*^2^) statistic.^[28]^ The *I*^2^ statistic of less than 50% indicates a low level of statistical heterogeneity, and that of 50% or more will be considered substantial statistical heterogeneity. If substantial heterogeneity is identified, we will report it and explore possible causes using sensitivity analysis and subgroup analysis.

#### 2.3.8 Assessment of reporting biases

A funnel plot will be constructed and examined to assess publication bias and possible small study biases if the group include more than 10 trials.^[29]^ The results will be interpreted carefully based on several explanations for funnel plot asymmetry.

#### 2.3.9 Data synthesis

If 2 or more eligible RCTs are identified, meta-analysis will be performed with Review Manager 5.3. All tests are 2-tailed, and *P* < .05 is considered statistically significant. Whether a fixed effects model or a random effects model will be used depends on the results of *I*^2^ test for heterogeneity. If *I*^2^ test <50%, a fixed effects model will be used to pool the data. If *I*^2^ test ≥50%, a random effects model will be used for data analysis instead. Subgroup analysis and sensitivity analysis will be performed to explore the causes of heterogeneity. If meta-analysis is not applicable, we will conduct a systematic narrative synthesis providing information to summarize and explain the characteristics and findings of the included studies.

#### 2.3.10 Subgroup analysis

We plan to carry out the following subgroup analyses if possible: comparison between acupuncture and no treatment, placebo or sham acupuncture, LM, western medicine treatment; comparison between acupuncture plus another therapy and the same therapy alone; different types of prediabetes, including IFG, IGT, or both conditions. We will use the formal test for subgroup interactions in Review Manager 5.3.

#### 2.3.11 Sensitivity analysis

Sensitivity analysis will be performed to explore the effects of trial risk of bias on primary outcomes if possible. In the analysis, we will exclude lower quality trials and repeat the meta-analyses to examine whether the quality of included studies influences the pooled results.

#### 2.3.12 Grading the quality of evidence

The Grading of Recommendations Assessment, Development, and Evaluation (GRADE) methodology will be used to assess the quality of the evidence and risk of bias with GRADEprofiler (GRADEpro) V.3.6 software. The assessment will be adjudicated into 4 levels: high, moderate, low, or very low.

## 3 Discussion

**Figure 1:**
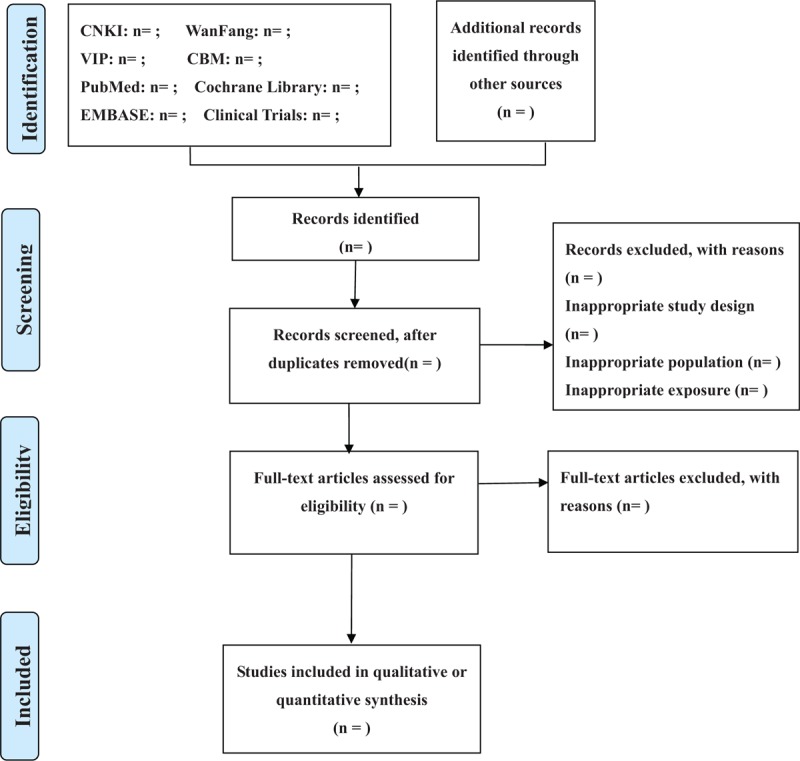
Flow diagram of study selection.

Prediabetes is prevalent and significantly increases lifetime risk of progression to T2DM.^[1,2,4–6]^ Numerous landmark trials have demonstrated that LM and pharmacologic interventions could delay or prevent incident diabetes among individuals with prediabetes, but it is not easy for patients to maintain rigorous and sustained LM for the long term and none of the tested pharmacologic interventions have been widely adopted as components of routine clinical care.^[7–19]^ There are an increasingly number of studies on acupuncture for prediabetes published with inconclusive results in recent years. Acupuncture may be a useful treatment for prediabetes. Therefore, it is essential to perform a systematic review and meta-analysis to assess the effect of acupuncture in preventing or delaying the development of T2DM. The flow diagram of this systematic review is shown in Figure 1. This review will be helpful to clinicians treating prediabetes and may provide evidence for researchers. Patients with prediabetes may also benefit from potential alternative interventions.

However, this systematic review will have some limitations. Only studies published in English and Chinese will be searched and included because of language barriers, so a language bias may exist. High heterogeneity may also arise from the various evaluation standards from different acupuncture therapies. Nevertheless, this systematic view should help further expand our understanding of acupuncture treatments in prevention of T2DM.

The PRISMA-P (Preferred Reporting Items for Systematic review and Meta-Analysis Protocols) checklist of this protocol is presented in PRISMA-P checklist.

## Author contributions

QN conceived of the study, supervised LS to perform this review. The manuscript was drafted by LS and LF. LS and LF developed the search strategy. YY and XL will independently screen the potential studies and extract data. MZ and YZ will assess the risk of bias and perform data synthesis. QN will arbitrate any disagreement and ensure that no errors occur during the review. LS and LF contributed equally to this work and are co-first authors. All review authors critically reviewed, revised and approved the subsequent and final version of the protocol.

**Conceptualization:** Qing Ni.

**Data curation:** Yanan Yang, Xiaowen Li, Meizhen Zhang, Yueying Zhang.

**Methodology:** Liwei Shi.

**Project administration:** Liwei Shi.

**Supervision:** Qing Ni.

**Writing – original draft:** Liwei Shi, Ling Feng.

**Writing – review & editing:** Liwei Shi.

## Supplementary Material

Supplemental Digital Content
