# Supplementary material for: Prevention of type 2 diabetes mellitus with acupuncture: Protocol for a systematic review and meta-analysis
Source: Medicine (Baltimore). 2018 Nov 30;97(48):e13355. doi: 10.1097/MD.0000000000013355 (PMC6283081; doi:10.1097/MD.0000000000013355)
Supplement: Supplemental Digital Content [file medi-97-e13355-s001.pdf]

Prevention of type 2 diabetes mellitus with acupuncture: protocol for a systematic review and meta-analysis

Relevant studies will be obtained from English database CENTRAL, PubMed, EMBASE, clinical trials; Chinese Database of Chinese National Knowledge Infrastructure Database (CNKI), Chinese Biomedical Literature Database (CBM), Chinese Scientific Journal Database (VIP), Wan Fang Database.

Search Strategy for PubMed

#1 acupuncture [mh]
#2 acupuncture therapy [all fields]
#3 acupuncture treatment [all fields]
#4 acupoints [mh]
#5 acupuncture points [all fields]
#6 electroacupuncture [mh]
#7 ear acupuncture [mh]
#8 auricular acupuncture [all fields]
#9 scalp acupuncture [all fields]
#10 fire needling [all fields]
#11 plum blossom needle [all fields]
#12 elongated needle [all fields]
#13 intradermal needling [all fields]
#14 dermal needling [all fields]
#15 dry needling [all fields]
#16 manual acupuncture [all fields]
#17 body acupuncture [all fields]
#18 #1 or #2 or #3 or #4 or #5 or #6 or #7 or #8 or #9 or #10 or #11 or #12 or #13 or #14 or #15 or #16 or #17
#19 prediabetes [mh]
#20 impaired fasting glucose [mh]
#21 IFG [all fields]
#22 impaired glucose tolerance [mh]
#23 IGT [all fields]
#24 impaired glucose regulation [mh]
#25 IGR [all fields]
#26 diabetes [all fields]
#27 prevention [all fields]
#28 hyperglycemia [all fields]
#29 normoglycemia [all fields]
#30 #19 or #20 or #21 or #22 or #23 or #24 or #25 or #26 or #27 or #28 or #29
#31 #18 and #30
#32 randomized controlled trial [pt]
#33 controlled clinical trial [pt]
#34 randomized [tiab]

#35 placebo [tiab]
#36 drug therapy [sh]
#37 randomly [tiab]
#38 trial [tiab]
#39 groups [tiab]
#40 #32 or #33 or #34 or #35 or #36 or #37 or #38 or #39
#41 animals [mh] not (humans [mh] and animals [mh])
#42 #40 not #41
#43 #31 and #42
